# Peroral cholangiopancreatoscopy-guided diagnosis and treatment of an intramural bile duct stone complicated with immunoglobulin G4-related cholangitis: a case report with video

**DOI:** 10.1055/a-2779-5197

**Published:** 2026-01-30

**Authors:** Sichao Wen, Haiyong Long, Ping Wang, Wenguang Yang, Yuhong Ren, Bin Yang, Mingwen Guo

**Affiliations:** 1 Department of Gastroenterology, Qionglai Medical Center Hospital, Qionglai, China

The development of peroral cholangiopancreatoscopy (POCPS) has enabled the super-minimally invasive direct visualization diagnosis and treatment of biliopancreatic diseases. Over the past half-century, continuous advancements in related equipment and accessories have promoted the evolution of POCPS-guided super-minimally invasive therapies 1 . For diagnosis, direct endoscopic visualization of unexplained biliopancreatic abnormalities combined with targeted biopsy has become an effective approach. In terms of treatment, POCPS-guided laser or electrohydraulic lithotripsy provides a safe and effective option for refractory bile duct and pancreatic stones 2 . Herein, we report a rare case of an intramural bile duct stone successfully managed by POCPS, with subsequent pathological confirmation of immunoglobulin G4-related cholangitis (IgG4-RC) from the resected bile duct wall tissue.

**Fig. 1:**
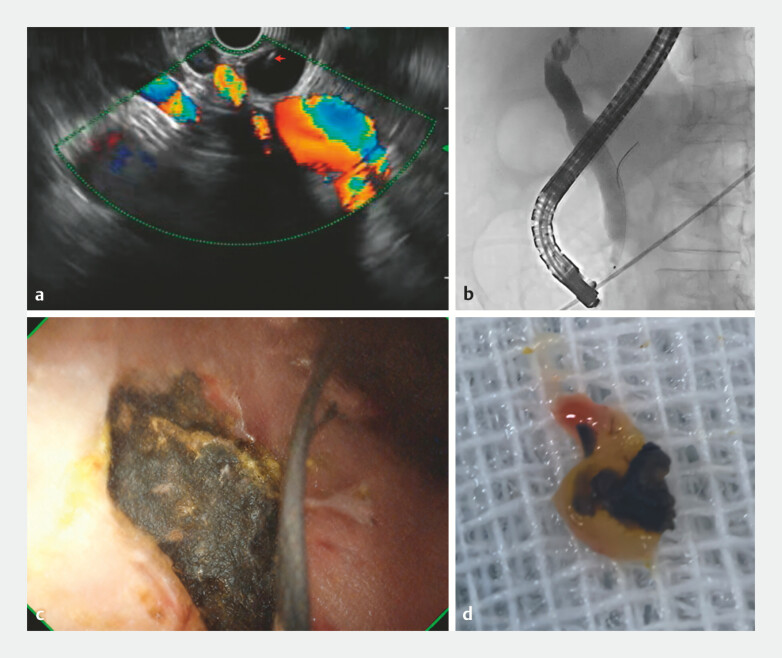
Peroral cholangiopancreatoscopy (POCPS)-guided visualization and retrieval of an intramural bile duct stone in a patient with immunoglobulin G4-related cholangitis. **a** EUS showing a tiny hyperechoic lesion in the bile duct, suggestive of a small stone. **b** ERCP was performed, and cholangiography showed no filling defects. **c** Further POCPS identified a 4-mm intramural stone in the distal bile duct. **d** POCPS-guided the successful retrieval of the stone and adjacent bile duct wall tissue. ERCP, endoscopic retrograde cholangiopancreatography; EUS, endoscopic ultrasound.

A 58-year-old woman presented to our department with recurrent abdominal pain and abnormal liver function for 3 years. Serum biochemistry showed mild elevations in transaminases, bilirubin, alkaline phosphatase, and gamma-glutamyl transferase. Computed tomography (CT) and magnetic resonance imaging (MRI) revealed a post-cholecystectomy status, with irregular morphology of the common hepatic duct and intrahepatic bile ducts but no evidence of stones. Subsequent endoscopic ultrasound (EUS) demonstrated a tiny hyperechoic lesion in the bile duct, suggestive of a small stone ( Fig. 1 **a** ). After obtaining informed consent, endoscopic retrograde cholangiopancreatography (ERCP) was performed, but cholangiography showed no filling defects ( Fig. 1 **b** ). Further POCPS examination identified a 4-mm intramural stone in the distal bile duct ( Fig. 1 **c** ). The stone and a portion of the surrounding bile duct wall tissue were successfully retrieved under POCPS guidance ( Fig. 1 **d** , Video 1 ). Postoperative pathology confirmed IgG4-RC, which was further supported by a marked elevation in serum IgG4 levels.

This video demonstrates the peroral cholangiopancreatoscopy (POCPS)-guided diagnosis and treatment of a 58-year-old woman with an intramural bile duct stone complicated by immunoglobulin G4-related cholangitis.Video 1

Intramural bile duct stones are extremely rare. EUS exhibits higher sensitivity than CT or MRI for their detection, but definitive diagnosis relies on direct POCPS visualization, which also allows simultaneous stone removal. IgG4-RC may be the underlying cause of intramural stone formation in this case. POCPS provides a safe and effective diagnostic and therapeutic modality for biliopancreatic diseases.

Endoscopy_UCTN_Code_CCL_1AZ_2AN
